# Evaluation of peptide-rich root extracts of *Calliandria portoriscensis* (Jacq.) Benth (Mimosaceae) for in vitro antimicrobial activity and brine shrimp lethality

**DOI:** 10.1186/s12906-020-2836-6

**Published:** 2020-02-03

**Authors:** Omonike O. Ogbole, Nkiruka C. Ndabai, Toluwanimi E. Akinleye, Alfred F. Attah

**Affiliations:** 1 Department of Pharmacognosy, Faculty of Pharmacy, University of Ibadan, Ibadan, Nigeria; 2 Department of Pharmacognosy and Drug Development, Faculty of Pharmaceutical Sciences, University of Ilorin, Ilorin, Nigeria

**Keywords:** Host defence peptides, *Calliandra portoricensis*, Antimicrobial, Brine shrimp lethality assay

## Abstract

**Background:**

Several Host defence peptides (HDPs) are low molecular weight (< 50 amino acids residues) peptides detected in several ethnomedicinal plants and have particularly gained research interest in recent times. Due to their wide range of bioactivity, occurrence, abundance and ability to induce very little resistance, they hold promising potentials in drug development. This study investigated the presence of bioactive peptides in the roots of *Calliandra portoricensis* (CPr) (Mimosaceae) and evaluated its antimicrobial activity against gram-negative and gram-positive bacteria.

**Methods:**

The crude peptide extract was obtained and pre-purified on pre-loaded tube of RP-C_18_ solid phase cartridges (strata giga tube C18-E; 5 g, 20 mL, Phenomenex, Germany). Peptide enriched fraction was chemically analysed for arginine-rich/aromatic amino acid-rich peptides using a modified G-250 analytical stain and ninhydrin on thin layer chromatography (TLC) for a preliminary screening. Furthermore, MALDI TOF/TOF peptidomics was used to detect the presence and masses of the peptides. Extracts from CPr were used to test the ability to inhibit microbial growth using p-INT (Para-iodonitrotetrazolium violet) dye, with 0.1% gentamycin as positive control. The concentration that inhibits the growth of microorganisms by 50% (IC_50_) were determined. Toxicity of the two extracts was accessed using freshly hatched nauplii of *Artemia salina.* Data analysis were evaluated using Microsoft excel and GraphPad Prism5.

**Results:**

Low molecular weight (LMW) peptides were detected in CPr using TLC and MALDI-TOF MS. Generally, the extracts exhibited good inhibition (70–95%) against the gram-negative and gram-positive bacteria, except MRSA6 typed strain. Enhanced activity was observed in the pre-purified peptide fraction than in the methanol crude, except on MRSA6. The greatest antimicrobial inhibition by pre-purified peptide fraction was against MRSA22 (IC_50_ = 0.69 ± 0.33 μg/mL). The crude methanol extract (LC_50_ = 5.13 μg/mL) was slightly more toxic than the peptide extract (LC_50_ = 6.12 μg/mL).

**Conclusions:**

This is the first report on detection of bioactive LMW peptides in Mimosaceae family. These peptides appear to be rich in arginine and aromatic amino acids. The peptide extract, in its pre-purified form showed a lower Brine shrimp cytotoxicity and an enhanced antimicrobial activity against the tested gram-negative and gram-positive bacteria.

## Background

Microbial infectious diseases represent an important and common cause of morbidity and mortality among the world population, particularly in developing countries [1–3]. The menace caused by microbial infection is increasing largely due to constantly emerging drug resistance of microorganisms to the conventional antimicrobials [4, 5]. Bacterial multi-resistance to medications in the market is evidently linked to their genetic ability to acquire and transmit resistance to sensitive strains [6–8], hence, the necessary motivation to continuously discover and develop new antimicrobials. Consequently, pharmaceutical companies have recognized the need for change in the molecular structure of the existing antimicrobials [6, 9–12]. However, ethnomedicinal plants continue to provide rich and limitless source of diverse molecular structures for the discovery and development of novel drug leads [13–15].

Plants, in addition to their ability to produce defensive secondary metabolites in response to microbial attack, can also produce defence molecules such as antimicrobial proteins and peptides, digestive enzymes, as components of their innate immunity [16, 17].

Antimicrobial peptides (AMPs) are gene-encoded as precursor proteins, and are structurally classified into three groups: α-helical, β-sheet, and extended peptides which are rich in cysteine, arginine, lysine and proline. All AMP groups adopt a basic structural principle of amphipathic design, in which clusters of hydrophobic and cationic amino acids are segregated, allowing for their selective electrostatic interaction with anionic bacterial cytoplasmic membranes. This results in their disruption, as against eukaryotic membrane which has no net charge (zwitterionic lipids) [18, 19]. AMPs’ other mechanisms of action include ion channel formation, microbes entangling, disruption of many cytoplasmic processes (such as cell wall, protein and nucleic acid synthesis) and hindrance of the formation of bacterial biofilms [18, 20–22]. Multiple modes of action is the reason AMPs could induce minor resistance, and this has attracted the attention of pharmaceutical industries with the aim to develop potent AMP drugs [18, 23]. Antimicrobial peptides rich in basic amino acids have been found to be more soluble in aqueous media [20]. This property additionally aid their extraction and detection using the modified G-250 analytical stain on thin layer chromatography (TLC) plates [24, 25]. This results from the ability of G-250 stain to form a stable complex with aromatic and basic amino acids residues in proteins resulting in deep blue colour and bright blue colour for a G-250-peptide complex when modified G-250 stain is used [26].

Generally, the abundance of antimicrobial peptides (AMPs) in plants and their physiological roles as defensive molecules is being explored as a potential source of anti-infective agents or lead compounds [24, 27, 28]. There is therefore the need to explore more families of the angiosperm for the discovery of HDPs since tropical Africa hosts one of the world’s richest flora diversity.

*Calliandra portoricensis* (CP) (Jacq.) Benth is an ethnomedicinally important straggling perennial shrub belonging to the family Mimosaceae. Several of the traditional uses of CP have received scientific validation including antimicrobial, antiproliferative, Antiulcer and chemo preventive [29–33]. In Nigeria, the roots are traditionally important as an antimicrobial agent [34], in managing pain, ulcer and convulsions [35]. However, there has been no report on the detection and isolation of bioactive peptides from this plant. This study therefore presents the chemical detection of low molecular weight bioactive peptides in the root of *C. portoricensis* (Mimosaceae family) and the evaluation of their antibacterial activity and brine shrimp lethality using crude methanolic and pre-purified Arginine-rich Peptides fraction (ARP).

## Methods

### Preparation of the extracts

Fresh roots of *Calliandra portoricensis* were harvested at Kabba bush, Kabba bunu local area, Kogi state, North Central, Nigeria. The plant was identified by Mr. T. K. Odewo of Forestry Research Institute of Nigeria (FRIN) and voucher specimen deposited at Forestry Herbarium Ibadan (FHI) with voucher number FHI 109672. The roots were washed, peeled, chopped, air dried, pulverized, and stored in an air-tight bag. In prospect of the target peptide molecules, standard extraction methods [36–40] were followed. For peptide extraction, dried and powdered plant material was extracted using equal volumes of methanol: dichloromethane under continuous agitation for 18–24 h at room temperature. Distilled water was added to the extraction system and vortexed. The upper aqueous layer was decanted and concentrated using the rotary evaporator (Buchi, Switzerland) to remove methanol prior to C_18_ flash pre-purification. The aqueous extract was purified on RP-C_18_ solid-phase with C18-parked Cartridges (Strata Gigatubes C_18_-E; 5 g, 20 mL, Phenomenex, Germany). Following initial preconditioning, equilibration and sample application, bound peptides were washed with 20% solvent B (90%v/v acetonitrile, 0.08% v/v trifluoroacetic acid) and finally eluted with 20 mL of 80% solvent B. Cryodesiccation of eluted fractions was achieved with the freeze drying equipment after pipetting out 200 μl each eluate for preliminary MALDI-TOF MS analysis of Peptide-like peaks. The root powder was equally extracted in methanol to obtain crude methanol extract.

### Preparation of the modified G-250 dye and ninhydrin reagent

The G-250 dye was prepared according to the modified method described by [25]. Two hundred milligram of G-250 was dissolved in 40 mL ethanol. Then, 320 mL of 50% ethanol in distilled water was prepared and 40 mL of orthophosphoric acid was added. The ninhydrin reagent was 0.2 g ninhydrin in 100 mL of ethanol.

### Chemical detection by TLC and MALDI TOF MS

The pre-purified peptide extract was chemically detected using Thin Layer Chromatography (TLC). A modified method previously described by [25, 41] and also used by [24] was adopted for the TLC chemical detection. Pre-coated TLC plates (G_254_ MERCK, Germany) were used. The solvent system used was n-butanol: acetic acid: water (3:1:1 v/v). The solvent reconstituted aqueous peptide fraction obtained from plant samples were spotted on the TLC plate and developed in the above-mentioned solvent system. The developed plates were allowed to dry, and sprayed with freshly prepared G-250 modified stain and Ninhydrin to detect the presence of circular and linear peptides, respectively. The sprayed plates were allowed to dry and viewed under UV at 245 nm and 365 nm. For MALDI TOF MS detection, 0.5 μL each of eluted fraction obtained from the C_18_ pre-purification as described above was mixed well with 3 μL of CHCA (alpha cyano hydroxyl cinnamic acid) matrix, spotted on the MALDI target plate and dried away from light. Acquired spectra were processed using the 4800 Analyzer. The freeze dried fractions were reconstituted and again analysed for peptide mass peaks as described above [40].

### Brine shrimp (*Artemia salina*) lethality assay (BSLA)

The assay was carried out according to the method of [42]. After hatching, nauplii were collected by dropping pipette. Twenty milligram each of the extracts (crude methanol and peptide) were dissolved in 2 mL of sea water. The stock solution for the methanol crude (1000 μg/mL) and peptide (500 μg/mL) extracts were further serially diluted to ranging concentrations of 1000 to 1 μg/mL and 500 to 4 μg/mL, respectively. The diluted test solutions of the peptide and crude were added to the test tubes in different concentrations. Then, ten (10) active brine shrimp (*nauplii*) were transferred into each of these vials using Pasteur pipette. Triplicates of each of the dose levels were prepared, using seawater as control. Number of survivors and deaths were recorded after 24 h.

### Antimicrobial assay

#### Preparation of extract stock solution

Methanol crude and pre-purified peptide extracts / Arginine-rich Peptides (20 mg) each from *C. portoriscensis* root (CPr) were dissolved in 2 mL of dimethylsulphoxide (DMSO) to make 1 mg/ml of stock and stored at 4 °C until use.

#### Microbial cultures

Gram-negative strain, *Escherichia coli* (ATCC 25922), and Gram-positive Strains Methicillin-Resistant *Staphylococcus aureus* (MRSA), *Staphylococcus aureus* (ATCC 6538), MRSA 6 and MRSA 22 were maintained on Nutrient agar, Mannitol salt agar (MSA), Muller-Hinton agar (MHA) respectively. MRSA 6 and MRSA 22 has been genotypically identified by the detection of *mec*A gene using established procedure [43]. A single colony of each organism was inoculated into 5 mL Tryptic Soy Broth (TSB) for preparation of bacterial culture. All microbes were sub-cultured from the original culture and incubated overnight at 37 °C. The bacteria were obtained from the Pharmaceutical Microbiology Department, University of Ibadan, Ibadan, Nigeria.

#### Standard antibiotic

The standard drug, 0.1% gentamycin was used as the positive control.

##### Qualitative antibacterial assay- solid media growth inhibition assay

The crude and pre-purified peptide extracts of CPr were mixed with SDS-polyacrylamide gels (for easy diffusion of the extracts), which were cut and deposited into a Petri dish. A single bacterial colony was inoculated into 5 mL TSB and incubated at 37 °C until it grew up to achieve a cell density equivalent to 0.5 Mc Farland. A 10 μL-aliquot of bacterial cultures was inoculated into 10 mL sloppy agar (0.5% agar) kept at a temperature of 50 °C. The mixture was homogenized and poured onto the plate. SDS-gel containing markers and buffer were used as negative controls, while Gentamicin (0.1%) was used as positive control. The plates were incubated at 37 °C during 24 h and the growth inhibition was analysed.

##### Quantitative antibacterial assay- minimum inhibitory concentration (MIC) determination

MIC was determined by the broth micro-dilution method using sterile 96-well microtiter plate [44]. Stock solutions of the CPr (methanol crude and pre-purified peptide) extracts (10 mg/mL) were diluted with culture TSB to a working concentration of 200 μg/mL. Serial two-fold dilutions of the working concentrations were made in the microtitre plate using TSB (in triplicate), to obtain concentrations ranging from 12.5 to 200 μg/mL. All controls including sterility control (broth and plant extract), control (containing culture broth and DMSO, without antimicrobial substance), positive control (Gentamycin), and the negative control (broth and organism) were as well distributed in the 96-well plates.

Each test and control well was inoculated with 50 μL (0.5 McF) of each bacterial suspension. All experiments were performed in triplicates and the micro-dilution plates were incubated at 36 °C for 24 h. After 24 h, 20 μL of 0.5 mg/mL *p-*INT alcoholic solution (p-iodonitrotetrazolium violet, Sigma®) was added to the plates. The plates were further incubated at 36 °C for 30 min. Wells with colour change from yellow to pinkish red indicated bacterial or microbial growth. Bacterial growth was formally detected by optical density (ELISA reader, CLX800-BioTek Instruments), and the MIC values were defined as the lowest concentration of each extract, which completely inhibited microbial growth.

### Statistical analysis

The 50% inhibitory concentration (IC_50_) for each extract was calculated from concentration-effect curves after non-linear regression analysis using GraphPad prism5 software.

## Results

### TLC chemical detection and MALDI TOF MS analysis

**Fig. 1:**
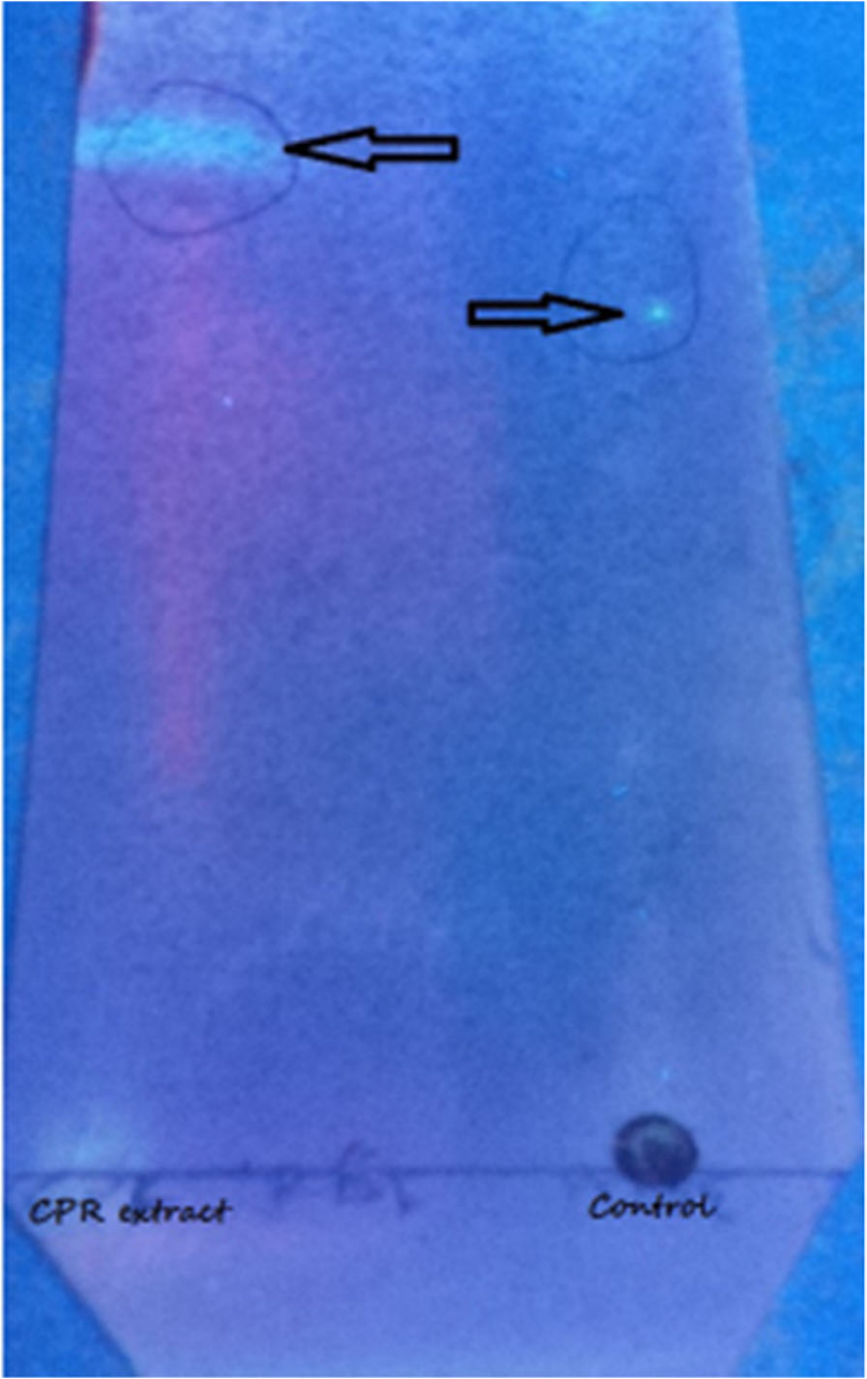
Chromatogram of peptide-rich aqueous fraction showing the presence of bioactive peptides at 360 nm. Solvent system: n-butanol: acetic acid: distilled water (3:1:1). Control: Arginine-containing cysteine-rich cyclotide extracts from the Nigerian plant *Rinorea dentata* [24].

*Calliandra portoriscensis* root (CPr) peptide-rich aqueous extract produced a bright blue colouration on the TLC plate (Fig. 1) upon reaction with the sprayed modified G-250 stain indicating the presence of arginine-rich or other basic amino acid-rich peptides as shown in Fig. 1. An additional spectra file shows the detail peptide distribution following MALDI TOF MS analysis (see Additional file 1).

### Brine shrimp lethality assay (BSLA)

The peptide-rich and methanol crude extracts were toxic to brine shrimp. However, the crude methanol extract of *C. portoricensis* was slightly more toxic (LC_50_ = 5.13 μg/mL) compared to the Arginine-rich Peptide (ARP) extract (LC_50_ = 6.12 μg/mL).

### Antimicrobial activity

The activities of the methanol crude and pre-purified peptide extracts tested on both Gram-positive and Gram-negative bacteria are compared, the results are displayed in Tables 1 and 2. *Staphylococcus aureus* was most susceptible to the peptide extract (IC_50_ = 0.69 ± 0.33 μg/mL) than the crude methanol extract (IC_50_ = 13.27 ± 0.00 μg/mL) while the crude methanol was more active on *Escherichia coli* than the peptide extract. The IC_50_ is defined as the concentration that inhibits the growth of test organisms by 50%. The resistance of MRSA 6 to the peptide extracts has been further demonstrated by the calculated high MIC value of 50 μg/mL (Table 2) and high IC_50_ value > 140 μg/mL. The IC_50_ value of the positive control (Gentamycin) against the tested bacteria ranges between 20.0 to 28.0 μg/mL, except for MRSA 6 in which case it was found to be 240 μg/mL.

**Table 1:** IC_50_ values of the crude and pre-purified peptide-rich extract from the root of *Calliandra portoricensis*

Extract	MRSA22	MRSA6	SA	*E. coli*	MRSA
	(μg/mL)				
Methanol crude	8.30.00 ± 0.00	760.00 ± 0.58	13.27 ± 0.00	6.31 ± 0.00	0.73 ± 0.00
Peptide Extract	13.27 ± 0.00	145.00 ± 0.00	0.69 ± 0.33	30 ± 0.33	1.17.00 ± 0.00
Gentamycin 0.1%	28.00 ± 0.00	240.00 ± 0.00	22.00 ± 0.33	20 ± 0.33	20.00 ± 0.33

*S.A Staphylococcus aureus* (ATCC 6538)*, MRSA* Methicillin Resistant *Staphylococcus aureus, E. coli Escherichia coli* (ATCC 25922), *Gent* Gentamycin,

SEM ± = Standard Error of the Mean

**Table 2:** The Minimum Inhibitory Concentration (MIC) values of the crude methanol extract and pre-purified peptide-rich extract of *Calliandra portoricensis* root

Extract	MRSA22	MRSA6	SA	*E. coli*	MRSA
	(μg/mL)				
Methanol Crude	12.25	50	12.5	25	25
Peptide extract	12.25	50	25	25	25
Gentamycin (0.1%)	≤ 12.5	≤ 12.5	≤ 12.5	≤ 12.5	≤ 12.5

## Discussion

Bioactive peptides, in particular, plant antimicrobial peptides (now preferably called host defence peptides) hold promising potentials in drug development due to their wide range of bioactivity, occurrence, diversity, abundance and ability to induce very little resistance [45]. Bioactive peptide-based drugs could present several potential merits over currently used antibiotics in the post antibiotic era. Several of the peptides have shown synergistic effect when applied in combination with standard antibiotics making them amenable for use in multidrug target approaches to optimise bioactivity and circumvent resistance of the pathogens [45].

Antimicrobial peptides containing basic amino acid side chains such as arginine, and strengthened by two or more disulphide connectivity have increasingly attracted scientific attention due to their potential for biologically active peptide drug discovery and development [46]. The presence of these amino acids tends to increase their binding interactions ultimately leading to improved bioactivity. Several of these bioactive peptides additionally have aromatic acid side chains such as phenylalanine, tyrosine and tryptophan which contribute both to their bioactivity as well as assist in detection in their pre-purified form [24, 47, 48]. The MALDI-TOF MS spectra analysis of the C_18_ pre-purified peptide mixture obtained from *C. portoricensis* indicates the presence of several peptide masses which observed antibacterial activity might be additive or synergistic owing to the fact that only peptide mixture and crude extract was used during this study (Fig. 1). The enhanced activity of the pre-purified peptide extract in inhibiting Gram-negative *E. coli* may have resulted from the presence of side chains such as arginine, lysine, histidine or aromatic acids which were detected during the chemical analysis. Arginine- and lysine-rich cationic side chains form around 20% of plant’s antimicrobial peptides (AMPs). The presence of aromatic side chains as well as polar cationic arginine and lysine residues plays an important role in the bioactivity and physico-chemical properties of AMPs by facilitating their solubilisation in aqueous media. Generally, the IC_50_ and MIC values validate a better activity of the pre-purified peptide fraction than the crude extract on both Gram-negative and Gram-positive bacteria (Table 1), except for MRSA 6.

Arginine-containing and aromatic acid-rich bioactive peptides from plants used in traditional medicine have been well reported in literature [49, 50] while synthetic analogues have shown tremendous activity against pathogenic bacteria and have received patent approval for clinical use [51]. These classes of peptides rich in basic and aromatic amino acids have demonstrated a superior antimicrobial activity against several pathogens including *Mycobacterium tuberculosis*, Salmonella Typhimurium, *Enterococcus faecalis* and fungi *Candida albicans*. Aside their antimicrobial potentials, the documented wide and varied bioactivity (including antibiofilm agents, immune modulators, and anti-inflammatories) of these peptides resulted in a more representative name - Host Defence Peptides (HDP) as opposed to the traditional name called antimicrobial peptides (AMPs) [52]. Host defence peptides with interesting antimicrobial activity have been characterised from many plants used in traditional medicine [53–55] while several others have been patented for use as peptide-based antimicrobial therapeutics [50, 51].

*Artemia salina* mortality, when exposed to chemically unknown botanical extracts/drugs, is considered a useful tool for the preliminary assessment of toxicity and/or pharmacology [56, 57]. Further exploration of these extracts with more sensitive cytotoxicity assays can lead to the isolation of potent cytotoxic compounds that can be harnessed into anticancer drug leads. The brine shrimp lethality assay (BSLA) of the crude methanol extract has earlier been reported [30]. The BSLA is very useful tool for studying bioactive compounds from plant extracts [56, 57]. Similarly, the toxicity of *C. portoricensis* peptide extract against *Artemia salina* suggest the potential of the peptides as anticancer molecules should there be discrimination between normal cells and cancer cells. This informed guess is consistent with the traditional application of the plant in the management of breast cancer in Southern Nigeria [58]. toxicity to tumour cell lines of the prostate [59] and chemo-prevents mammary gland toxicity in rodents [60]. All these reports provide an evidenced-based traditional application of the root of *C. portoricensis* in the management of various ailments including those caused by microbial pathogens or cancer-related disease burdens. Extracts of the plant that have shown anti-infective and anticancer potentials are aqueous/polar fraction which represents the peptide-rich fraction. However, this work is the first report on the detection of bioactive peptides in *C. portoricensis*. The brine shrimp toxicity of the peptide fraction may also be indicative of the synergistic activity of several peptides present in the extract [45].

## Conclusions

This study presents the first report on the presence of arginine/lysine/histidine-rich bioactive peptides in Mimosaceae, as well as the antimicrobial activity of the peptide enriched eluates against the gram-negative and gram-positive bacteria. MRSA typed 6 strain was found to have low sensitivity to the tested extracts and the standard drug (Gentamycin). The enriched peptide extract of *C. portoricensis* root showed enhanced antimicrobial activity than the crude methanol extract suggesting the activity of the antimicrobial peptides. This work has documented the identification of bioactive peptides containing basic amino acids in the study plant. Further work is on-going to characterize the bioactive peptides detected in the plant.

## Supplementary information

**Additional file 1.** MALDI TOF MS data showing monoisotopic mass/charge ratios for the peptide-rich pre-purified aqueous extracts of *Calliandra porturescensis* collected from South-west Nigeria. Identified peptide masses ranges from 2.0 KDa to 2.6 KDa.

## Data Availability

All data generated or analysed during this study are included in this article (and its supplementary information files), the materials and data of our study are available to other researchers upon request.

## Abbreviations

AMPs: Antimicrobial peptides
BSLA: Brine shrimp lethality assay
CPr: *Calliandra portoricensis root*
DMSO: Dimethylsulphoxide
ELISA: Enzyme-linked immunosorbent assay
FHI: Forestry Herbarium Ibadan
FRIN: Forestry Research Institute of Nigeria
HDPs: Host defence peptides
MALDI-TOF MS: Matrix-assisted laser desorption ionization-Time of Flight Mass spectrometry
MIC: Minimum inhibitory concentration
MRSA: Methicillin-resistant *Staphylococcus aureus*
*p-*INT: *para*-iodonitrotetrazolium
TLC: Thin layer chromatography
UV: Ultraviolet light
