# Supplementary material for: Evaluation of peptide-rich root extracts of Calliandria portoriscensis (Jacq.) Benth (Mimosaceae) for in vitro antimicrobial activity and brine shrimp lethality
Source: BMC Complement Med Ther. 2020 Feb 3;20:30. doi: 10.1186/s12906-020-2836-6 (PMC7076830; doi:10.1186/s12906-020-2836-6)
Supplement: Supplementary file 1 — Additional file 1. MALDI TOF MS data showing monoisotopic mass/charge ratios for the peptide-rich pre-purified aqueous extracts of Calliandra porturescensis collected from South-west Nigeria. Identified peptide masses ranges from 2.0 KDa to 2.6 KDa. [file 12906_2020_2836_MOESM1_ESM.docx]

Additional file 1


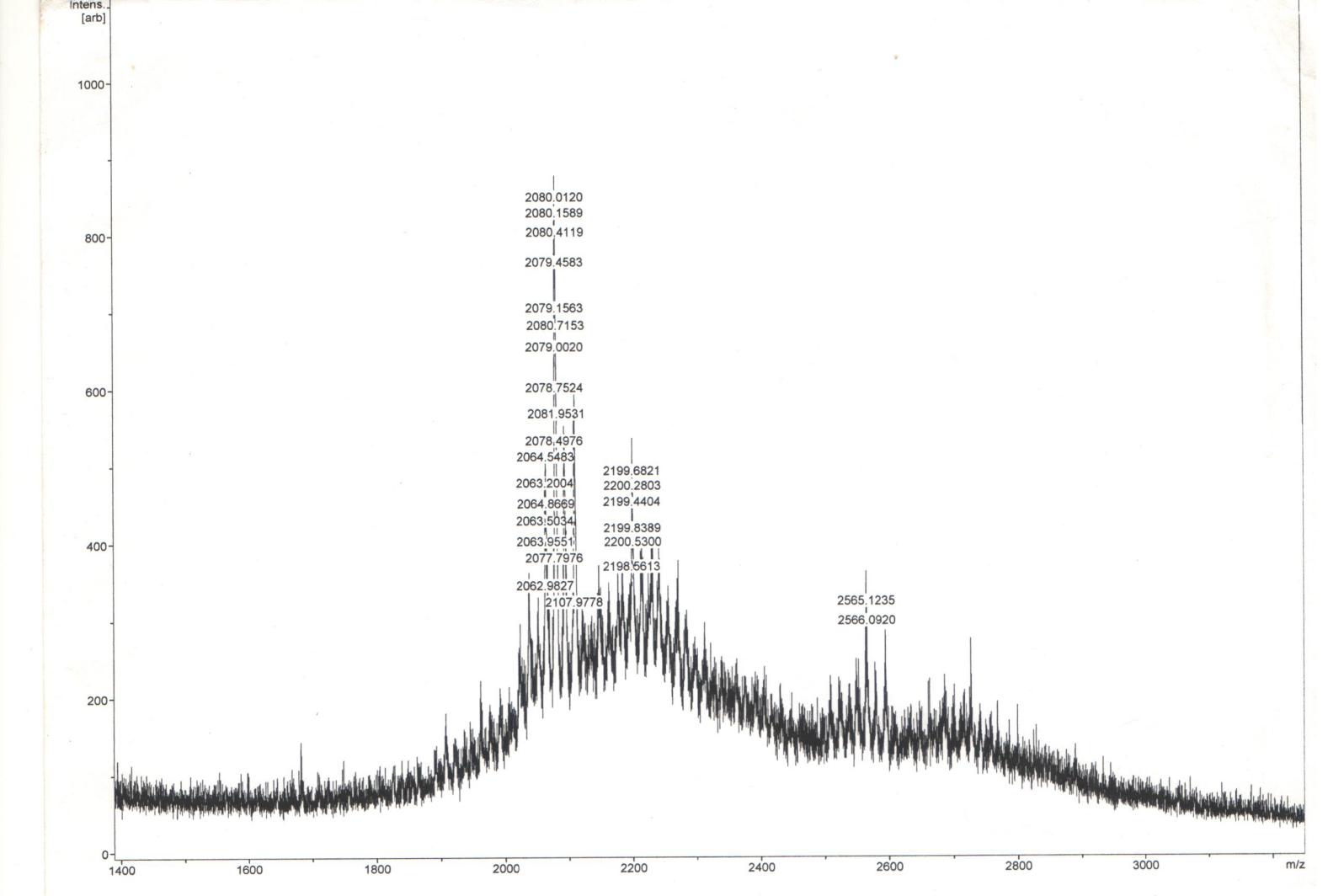


Relative intensity

mass/charge (m/z)

Additional file 1: MALDI TOF MS data showing monoisotopic mass/charge ratios for the peptide-rich pre-purified aqueous extracts of *Calliandra porturescensis* collected from South-west Nigeria. Identified peptide masses ranges from 2.0 KDa to 2.6 KDa.
